# MiR-30a and miR-379 modulate retinoic acid pathway by targeting DNA methyltransferase 3B in oral cancer

**DOI:** 10.1186/s12929-020-00644-z

**Published:** 2020-04-02

**Authors:** Shine-Gwo Shiah, Jenn-Ren Hsiao, Hsiao-Ju Chang, Yuan-Ming Hsu, Guan-Hsun Wu, Hsuan-Yu Peng, Sung-Tau Chou, Ching-Chuan Kuo, Jang-Yang Chang

**Affiliations:** 1 National Institute of Cancer Research, National Health Research Institutes, Miaoli, Taiwan; 2 Cancer Center, Wan Fang Hospital, Taipei Medical University, Taipei, Taiwan; 3 Ph.D. Program in Environmental and Occupational Medicine|, Kaohsiung Medical University, Kaohsiung, Taiwan; 4 Department of Otolaryngology, Head and Neck Collaborative Oncology Group, National Cheng Kung University Hospital, College of Medicine, National Cheng Kung University, Tainan, Taiwan; 5 Institute of Biotechnology and Pharmaceutical Research, National Health Research Institutes, Miaoli, Taiwan; 6 Division of Hematology and Oncology, Department of Internal Medicine, National Cheng Kung University Hospital, College of Medicine, National Cheng Kung University, Tainan, Taiwan

**Keywords:** microRNA, miR-30a, Retinoic acid (RA), DNA methyltransferase (DNMT), DNA methylation, Epigenetic regulation, Oral cancer, OSCC

## Abstract

**Background:**

Epigenetic silencing of retinoic acid (RA) signaling-related genes have been linked with the pathogenesis and clinical outcome in oral squamous cell carcinoma (OSCC) carcinogenesis. However, the precise mechanisms underlying the abnormal silencing of RA signaling-related genes in OSCC have not been well investigated.

**Methods:**

Using combined analysis of genome-wide gene expression and methylation profile from 40 matched normal-tumor pairs of OSCC specimens, we found a set of retinoid signaling related genes are frequently hypermethylated and downregulated in OSCC patient samples, including alcohol dehydrogenase, iron containing 1 (ADHFE1) and aldehyde dehydrogenase 1 family, member A2 (ALDH1A2), which are the important rate-limiting enzymes in synthesis of RA. The expression of ADHFE1 and ALDH1A2 in OSCC patients was determine by quantitative real-time PCR (qRT-PCR) and immunohistochemistry. The binding sites of miR-30a and miR-379 with DNA methyltransferase 3B (DNMT3B) were predicted using a series of bioinformatic tools, and validated using dual luciferase assay and Western blot analyses. The functions of miR-30a, miR-379, and DNMT3B were accessed by growth and colony formation analyses using gain- and loss-of-function approaches. Chromatin immunoprecipitation (ChIP) was performed to explore the molecular mechanisms by arecoline and 4-(methylnitrosamino)-1-(3-pyridyl)-1-butanone (NNK) treatment.

**Results:**

We demonstrated that deregulated miR-30a and miR-379 could represent a mechanism for the silencing of ADHFE1 and ALDH1A2 in OSCC through targeting DNMT3B. Ectopic expression of miR-30a and miR-379 could induce re-expression of methylation-silenced ADHFE1 and ALDH1A2, and lead to growth inhibition in oral cancer cells. Furthermore, the dysregulation of the miRNAs and DNMT-3B may result from exposure to tobacco smoking and betel quid chewing.

**Conclusions:**

Our results demonstrate that tobacco smoking and betel quid chewing could repress miR-30a and miR-379, which upregulate the DNMT3B expression, in turn, lead to the hypermethylation of ADHFE1 and ALDH1A genes, consequently, promote the oncogenic activity. These findings highlight the potential use of retinoids in combination with epigenetic modifiers for the prevention or treatment of oral cancer.

## Background

Oral squamous cell carcinoma (OSCC) is the most common cancer of the head and neck worldwide. Even though outstanding diagnostic and therapeutic improvements are available, mortality associated with OSCC is still extremely high [1, 2]. Therefore, it is urgent to identify reliable prognostic biomarkers for treatment failure, as well as to develop innovative drug targets for more effective and less toxic treatment. Recent evidence indicates that epigenetic alterations, apart from genetic alteration, have been linked with the pathogenesis and clinical outcome in OSCC carcinogenesis [3, 4]. The most common epigenetic alteration in OSCC is aberrant DNA methylation, which can silence gene expression and regulate biological processes, and play an important role in cancer initiation, progression, and metastasis [5, 6]. However, in the last decade, our understanding on how epigenetic alterations affect the tumor response and its clinical application in OSCC carcinogenesis is still limited.

Previously, we have conducted a global DNA methylation analysis of 40 OSCC primary tumor samples to Gene Expression Omnibus (GEO) database (Accession number GSE45238), which showed a specific signature of gene promoter methylation profile [7]. Among these genes, we found that a group of retinoic acid (RA) signaling-related genes are silenced by promoter hypermethylation in OSCC patients, including alcohol dehydrogenase, iron containing 1 (ADHFE1) and aldehyde dehydrogenase 1 family, member A2 (ALDH1A2). ADHFE1 can oxidize retinol to retinaldehyde, which is further metabolized to RA by ALDH1A2. ADHFE1 and ALDH1A2 are the rate-limiting enzymes and responsible for synthesis of RA, which exerts its biological functions, including cell differentiation, cell cycle arrest, and apoptosis under physiological and pathological conditions, through binding to nuclear RA receptors (RARs) that form heterodimers with retinoid X receptors (RXRs) to activate downstream target genes [8]. RA and related synthetic products are approved for the treatment of T-cell lymphoma and acute promyelocytic leukemia [9, 10], however, the chemopreventive and therapeutic effects of retinoids in solid tumors, including head and neck cancer, have failed to show significant advantage [11]. One of the possible mechanisms is that DNA methylation leads to the aberrant RA signaling [8]. Several studies have demonstrated the methylation-silenced expression of ADHFE1 and ALDH1A2 may be responsible for reducing RA levels and altered RA signaling [12–14]. The epigenetic silencing caused aberrant RA signaling could contribute both to tumor development and, consequently, to render cells retinoid resistant [15].

Recently, increasing evidence suggests that microRNAs (miRNAs) play important roles in the promoter methylation of CpG islands by targeting the DNA methylation machinery [16, 17]. Aside from miRNAs, epidemiological studies and experimental evidences suggest that many environmental chemical carcinogens, such as cigarette smoking and betel quid chewing, can also affect epigenetic mechanisms [18, 19]. However, the molecular links among the chemical carcinogens, miRNAs, and reduced ADHFE1 and ALDH1A2 remain unclear in OSCC. In this study, we demonstrated that the exposure of oral cancer cell lines to 4-(methylnitrosamino)-1-(3-pyridyl)-1-butanone (NNK, one of the major components of tobacco), or arecoline (Are, a major betel nut alkaloid), significantly reduced the expression of miR-30a and miR-379. Deregulated miR-30a and miR-379 could represent a mechanism for the silencing of ADHFE1 and ALDH1A2 in OSCC through modulation of DNA methyltransferase 3B (DNMT3B). These results highlight a role of miRNAs in the aberrant DNA methylation of OSCC and support a pharmacological rationale for the combination of RA and DNA methylation inhibitors in the prevention of oral carcinogenesis.

## Methods

### Cell lines and culture condition

Human oral keratinocytes (HOK) (ScienCell Research Laboratories, Carlsbad, CA, USA) were cultured in oral keratinocyte medium (ScienCell Research Laboratories) according to the manufacturer’s instructions. OSCC cells, including SCC-4, SCC-9, SCC-15, SCC-25, TW2.6, OEC-M1, OC-3, HSC-3, DOK, and YD-15, were routinely cultured as previously described [20, 21]. All cells were cultured at 37 °C in a 5% CO2 atmosphere and maintained in 10% fetal bovine serum (FBS, Kibbutz BeitHaemek, Israel). The carcinogen-transformed DOK cells were maintained [22] and established by Dr. Ching-Chuan Kuo’s laboratory (Institute of Biotechnology and Pharmaceutical Research, National Health Research Institutes of Taiwan). In brief, DOK cells were treated with non-toxic concentration of carcinogens (NNK: 10 μM; arecoline: 50 μM) for 12 months, and the carcinogen-transformed cells were selected and designated as NNK-L (NNK-tolerant) and Are-L (arecoline-tolerant) cells.

### Tissue specimens

Paired tumor specimens and their adjacent nontumorous epithelia were from OSCC patients as previously described [20]. The study protocol was reviewed and approved by the Institutional Human Experiment and Ethic Committee of the National Cheng Kung University Hospital (No: HR-97-100). These matched pairs of oral tumor/adjacent normal (T/N) tissues were grouped into two sets, a training set containing 40 samples for genome wide microarray study and a validation set containing 33 samples for ADHFE1, ALDH1A2, DNMT3B, and miRNAs quantitative-PCR analysis. For immunohistochemical study, 36 of matched pairs of oral T/N specimens for ADHFE1 staining and 38 of matched pairs of oral T/N specimens for ALDH1A2 staining were used. Clinical profiling of microarray data are available in Gene Expression Ommibus (GEO) under accession number GSE37991 for gene expression, GSE45238 for miRNA expression and GSE38823 for methylation analysis.

### Immunohistochemistry (IHC)

For immunohistochemical study, OSCC tissues were deparaffnized using xylene and then rehydrated through an ethanol series. Antigens were retrieved by autoclaving the slides in Dako retrieval buffer (Dako, Carpinteria, CA, USA). After cooling to room temperature, the slides were incubated with primary ADHFE1 antibody (Sigma-Aldrich, St. Louis, MO, USA) or ALDH1A2 antibody (Santa Cruz Biotechnology, Santa Cruz, CA, USA) at 4 °C overnight. Specific signals were then developed with LSAB+ kit (Dako) using diaminobenzidine as chromogen. Sections were then counterstained with hematoxylin and observed under light microscope. Tumor ADHFE1 and ALDH1A2 level were scored according to staining intensity as follows: 0, negative; 1, weak; 2, intermediate; and 3, strong. Two pathologists independently assessed all the scorings.

### RNA extraction, reverse-transcription PCR (RT-PCR)

Total RNA was extracted from OSCC cell lines using TRIzol reagent (Life Technologies, Gaithersburg, MD, USA) according to the manufacturer’s instructions. RNA concentration was checked by NanoDrop ND-1000 spectrophotometer (Thermo Fisher Scientific, Wilmington, DE, USA). For mRNA analysis, the cDNA was synthesized using random hexamer primers and SuperScript III reverse transcriptase (Invitrogen, Carlsbad, CA). Gene expression analyses were assayed on a Biometra T3000 thermocycler (Biometra GmbH, Gttingen, Germany) and GAPDH was used as a loading control. PCR products were subjected to electrophoresis on 2% agarose gel and visualized on UVP GDS-8000 Bioimaging System (UVP, Upland, CA, USA) with 0.01% of SYBRSafe (Invitrogen) inner staining.

### Quantitative real-time PCR (qPCR)

For miRNA analysis, the cDNA was synthesized using specific stem-loop RT primers and TaqMan MicroRNA Reverse Transcription Kit (Applied Biosystems, Carlsbad, CA, USA). q-PCR analysis was used to detect the ADHFE1, ALDH1A2, and DNMT3B using Omics Green EvaGreen q-PCR Master Mix (OMICS Biotechnology, New Taipei City, Taiwan) and the expression level of miR-30a and miR-379 using QuantiTect SYBR Green PCR System (Qiagen, Hilden, Germany), respectively, according to the manufacturer’s instructions on the ABI StepOnePlus Real-time PCR system (Applied Biosystems). GAPDH and RUN44 were used as the internal controls. All reactions were run in triplicate and relative expression levels were calculated as 2-△△CT after normalization with the internal control. All primers used for this study are summarized in Additional file 1: Table S1.

### Plasmids and transfection

The entire 3′-UTR of DNMT3B fragment, containing target sequences of miR-30a and miR-379, were PCR amplified and cloned into the pmirGLO firefly luciferase-expressing vector (Promega, Madison, WI, USA) according to the manufacturer’s instructions. The miR-30a and miR-379 binding site mutation vectors were also constructed by using Site-Directed Mutagenesis Kit (Stratagene, La Jolla, CA, USA), and all the constructs were verified by DNA sequencing. For miRNA expression, the pri-form miRNA sequence was amplified and subsequent cloned into the pLemiR miRNA expression vector (Open-Biosystem, Rockford, IL, USA). For transfection of the plasmids, cells were transiently transfected with 2 μg of plasmids using Lipofectamine 2000 (Invitrogen) according to the manufacturer’s protocol. The miRNA inhibitors (AM) and miRNA mimics (PM) were chemically modified RNA nucleotides and obtained from Ambion. The nucleotide transfection was performed using Lipofectamine RNAiMAX (Invitrogen) according to the manufacturer’s instructions.

### Immunoblotting

Protein extraction and western blotting were performed as previously described [23]. Equal amounts of protein lysates were separated by 10~12% SDS polyacrylamide gels and transferred to poly-vinylidene fluoride (PVDF) membrane (Pall Life Sciences, Glen Cove, NY, USA). Immunoblotting was performed with specific antibodies against DNMT3B (NB300–516; NOVUS Biologicals, Centennial, CO, USA). α-Tubulin (sc-23,950; Santa Cruz Biotechnology) was used as the internal control. Signals from HRP-conjugated secondary antibodies were visualized by enhanced chemiluminescence (ECL) detection system (PerkinElmer, Waltham, MA, USA) and chemiluminescence was exposed onto KodakX-Omat film (Kodak, Chalon/Paris, France).

### Luciferase reporter assay

OEC-M1 cells were transfected with 100 ng of DNMT3B 3′-UTR wild-type (wt-3′-UTR) or mutant form (mt-30a-3′-UTR or mt-379-3′-UTR) pmirGLO reporter plasmid and co-transfected with 20 nM of miRNA mimics (PM-30a or PM-379) or control oligonucleotide (PM-NC) using Lipofectamine RNAiMAX Transfection Reagent (Thermo Fisher Scientific, Wilmington, DE, USA) according to the manufacturer’s instructions. After 48 h, luciferase activities were detected using the Dual Luciferase Reporter Assay System (Promega) on the Orion L luminometer (Berthold, GmbH, Pforzheim, Germany), according to the manufacturer’s protocol. Renilla luciferase served as the control reporter for normalization.

### Cell proliferation assay

Cell proliferation was measured using 3-[4,5-dimethylthiazol-2-yl]-2,5-diphenyl tetrazolium bromide (MTT, Sigma-Aldrich) as previously described [20]. The optical density (OD) at 550 nm was measured using a 96-well plate SpectraMax 250 reader (Molecular Devices, Sunnyvale, CA, USA). For colony formation assay, cells (150 cells/well for OEC-M1 and 300 cells/well for SCC-15) were seeded into 6-well plates and cultured for 7 days at 37 °C in culture hood. Colonies were fixed, stained with 1% crystal violet, and counted under a microscope.

### Chromatin immunoprecipitation (ChIP)

ChIP assay was performed based on previous described [24]. For miRNA treatment, OEC-M1 cells were treated with control mimics (NC, 20 nM), or miRNA mimics (PM-30a or PM-379, 20 nM) for 48 h. For chemical treatments, DOK cells were treatment with vehicle control (DMSO, 10 nM), arecoline (50 μM) or NNK (10 μM) for 5 days. For demethylation assay, the wild type (Wt), Are-L and NNK-L DOK cells were treated with 5-aza-dC (5 μM) for 5 days. And then fixed with formaldehyde for cross-link chromatin associated proteins to genomic DNA, lysed and sonicated to generate DNA fragments between 200 to 1000 base pairs (confirmed by agarose gel electrophoresis). Then, the cell lysate were subjected to immunoprecipitation overnight by DNMT3B (ab2851, Abcam, Cambridge, MA, USA) antibody and consequently for PCR assay. Primers used for this study are summarized in Additional file 1: Table S1.

### Bioinformatics and statistical analyses

Group differences were analyzed by the two-tailed Student t test. All statistical analysis and graph presentation were performed using GraphPad Prism 5 Software Ver.5.01 (San Diego, CA, USA). Correlations between pairs of data were performed by parametric Pearson Spearman correlation analyses. A value of *p* < 0.05 was considered as statistically significant.

## Results

### Retinoid pathway is downregulated and methylated in oral cancer

**Fig. 1:**
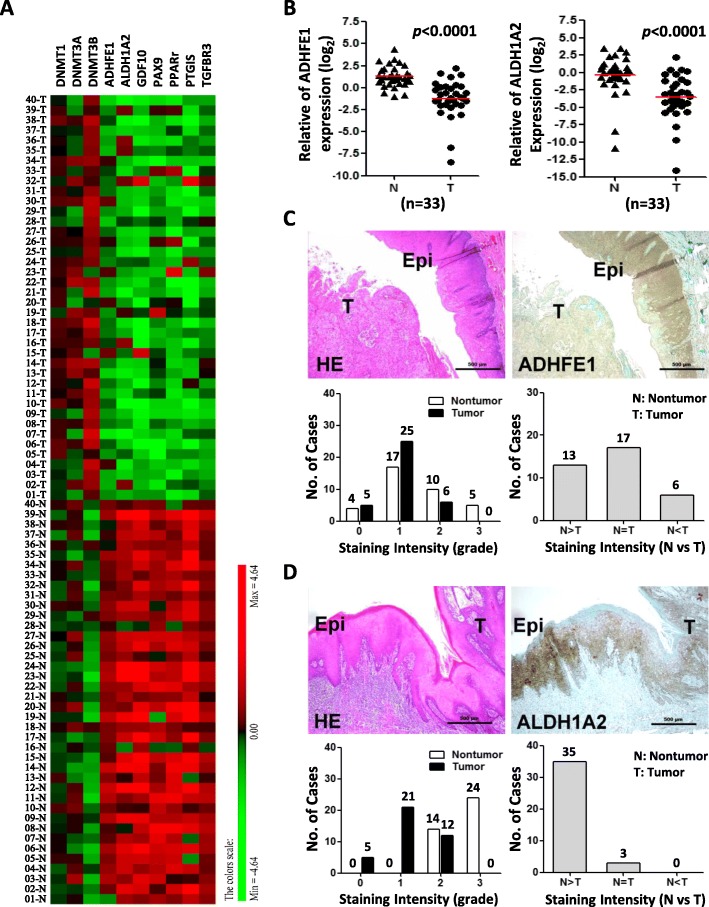
Downexpression of ADHFE1 and ALDH1A2 in OSCC tissues. **a** Heat map of the 10 differentially expressed mRNA, including DNMT families and retinoid signaling related genes in 40 OSCC tissue pairs (GSE37991). Red, overexpression; green, downexpression. **b** Validation of ADHFE1 and ALDH1A2 expression by qRT-PCR in another 33 of OSCC tumors (T) compared with their own adjacent normal tissues (N). Expression levels are expressed as the log_2_ ratios. Red lines are represented as group mean value; *p* < 0.0001. **c-d** Immunohistochemical staining of ADHFE1 and ALDH1A2 expression in human OSCC specimens. Top left, HE staining of a representative OSCC specimen, showing both adjacent normal oral epithelium (Epi) and underlying tumor nests (T) (scale bar, 500 μm). Top right, ADHFE1 or ALDH1A2 staining on the same region, demonstrating strong ADHFE1 or ALDH1A2 expression at the basal cell layer of the adjacent normal oral epithelium (Epi) and in the infiltrating tumor cells (T). Bottom left, results of ADHFE1 scoring grade for the 36 OSCC specimens pairs and ALDH1A2 scoring grade for the 38 OSCC specimens pairs. Bottom right, comparing of staining intensity between nontumor (N) and tumor (T) in the OSCC specimen

Upon analysis of gene expression array (GEO accession number GSE37991) and DNA methylation array (GEO accession number GSE45238) in 40 patients with OSCC, we found a set of retinoid signaling related genes, including ADHFE1, ALDH1A2, cellular retinol-binding protein 1 (CRBP1), paired box gene 9 (PAX9), growth differentiation factor 10 (GDF10), transforming growth factor beta receptor 3 (TGFBR3), and peroxisome proliferator-activated receptor gamma (PPARγ), were not only highly down-regulated but also methylated in OSCC cancer tissues compared with paired noncancerous tissues (Fig. 1a, Table 1). Because both ADHFE1 and ALDH1A2 are rate-limiting enzymes and participate in retinoid metabolism by oxidizing retinol to retinaldehyde to retinoic acid [25], therefore, we concentrated our attention on these two enzymes in following study. Using qRT-PCR to further validate the expression level of ADHFE1 and ALDH1A2, we found that ADHFE1 and ALDH1A2 levels were significantly downregulated in tumors than in corresponding normal samples (*p* < 0.0001)(Fig. 1b). Moreover, immunohistochemical analysis of the representative specimens of patients with OSCC demonstrated that the expression of ADHFE1 and ALDH1A2 expression was greater in nontumor squamous epithelium than in tumor tissues (Fig. 1c-d).

**Table 1:**
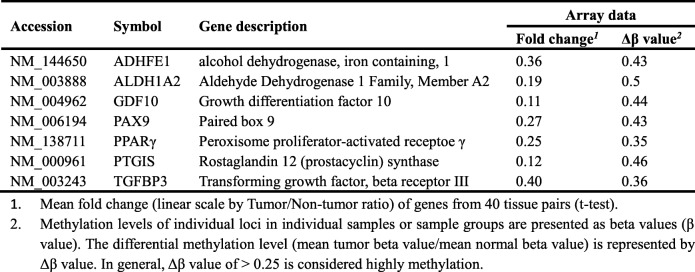
List of retinoid signaling related genes in OSCC

### DNMT3B involved in ADHFE1 and ALDH1A2 silencing

**Fig. 2:**
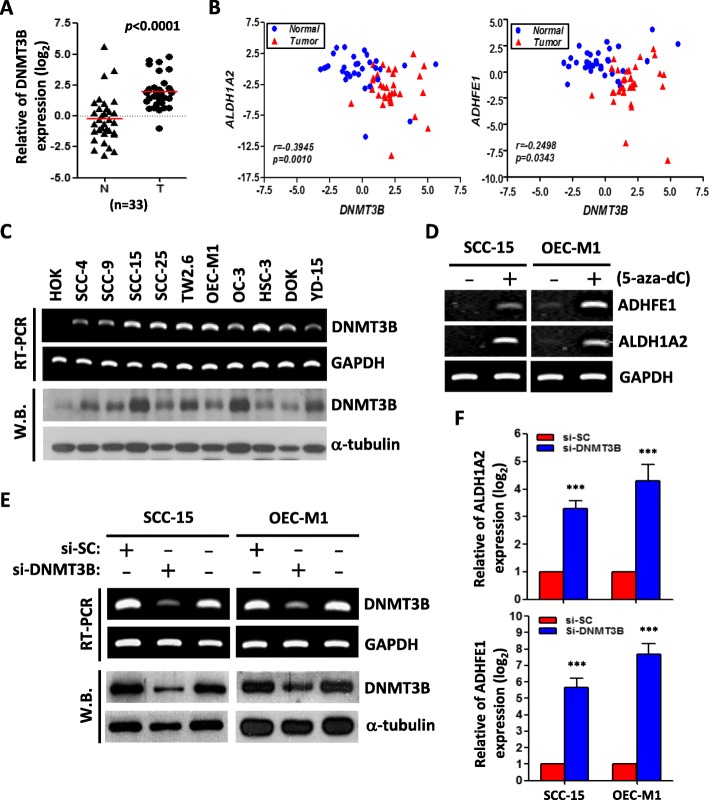
Silencing of ADHFE1 and ALDH1A2 through DNMT3B. **a** Relative DNMT3B expression levels in 33 of OSCC tumors (T) compared with their own adjacent normal tissues (N). **b** Correlation analysis of DNMT3B and ADHFE1 or ALDH1A2 in OSCC patients (*n* = 33) by qRT-PCR analysis. Pearson correlation coefficients and *p*-values were calculated as indicated. Red, tumor part; green, normal part. **c** Expression level of DNMT3B by RT-PCR and Western blot (W.B.) analysis in human oral keratinocyte (HOK) and OSCC cell lines. GAPDH and α-tubulin were used as internal control, respectively. **d** RT-PCR analysis of ADHFE1 and ALDH1A2 protein after 5-aza-dC (5 μM) treatment for 5 days. GAPDH was used as an internal control. **e** RT-PCR and Western blot analysis of DNMT3B in SCC-15 and OEC-M1 cells following DNMT3B knockdown (si-DNMT3B) or non-targeting siRNA control (si-SC) for 48 h. GAPDH and α-tubulin were used as internal control, respectively. **f** qRT-PCR analysis of ADHFE1 and ALDH1A2 expression after DNMT3B knockdown (si-DNMT3B) compared with siRNA control (si-SC) in SCC-15 and OEC-M1 cells. All data are presented as mean ± SD; ***p < 0.001

ADHFE1 and ALDH1A2 have been reported to be downregulated and hypermethylated in cancers [12, 13]. From our microarray data, we also found that DNMT1, DNMT3A and DNMT3B were overexpression in oral cancer (Fig. 1a). Next, we attempted to determine which DNMT member involved in methylation-mediated ADHFE1 and ALDH1A2 silencing. As shown in supplementary Fig. S1, DNMT3B is not only differentially expressed in tumor tissues (Additional file 2: Fig. S1a), but also has significant correlation with ADHFE1 and ALDH1A2 expression (Additional file 2: Fig. S1b). We further used an independent cohort of 33 OSCC tissues to validate the identified DNMT, and confirmed that DNMT3B displayed higher levels in OSCC tumors (Fig. 2a) and its expression negatively correlated with expression of ADHFE1 and ALDH1A2 with *p*-value< 0.05 (Fig. 2b). Consistent with this, western blotting and RT-PCR analysis revealed that DNMT3B levels are relatively higher in the most of OSCC cell lines when compared with the human normal keratinocyte HOK (Fig. 2c). To examine the role of aberrant methylation in deregulation of ADHFE1 and ALDH1A2 in OSCC, we evaluated the effect of the methylation inhibitor 5-aza-2-deoxycytidine (5-aza-dC) on ADHFE1 and ALDH1A2. We found that ADHFE1 and ALDH1A2 expression were increased by incubation with 5-aza-dC in both SCC-15 and OEC-M1 cells (Fig. 2d). To better study the methylation-dependent mechanism of ADHFE1 and ALDH1A2 change, we further manipulated the RNA interference to knockdown the DNMT3B expression (Fig. 2e). After the incubation of si-DNMT3B, the expression of ADHFE1 and ALDH1A2 mRNA were up-regulated in SCC-15 and OEC-M1 cells (Fig. 2f). These data indicated that ADHFE1 and ALDH1A2 can be modulated by epigenetic mechanism and DNMT3B plays a role in ADHFE1 and ALDH1A2 promoter methylation.

### MiR-30a and miR-379 directly target DNMT3b in OSCC cells

**Fig. 3:**
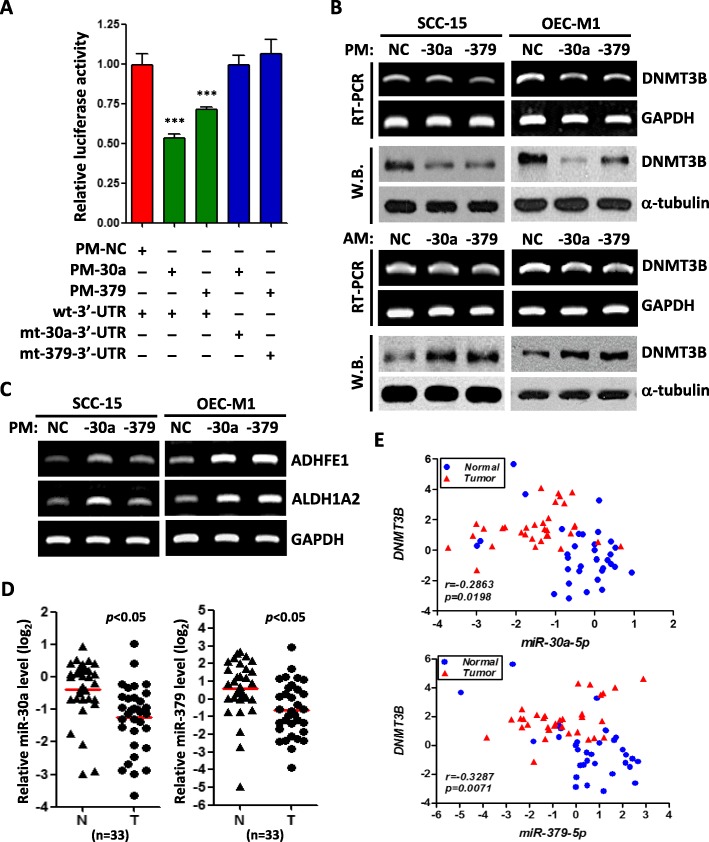
DNMT3B is a direct target of miR-30a and miR-379. **a** The effect of miRNA mimics (PM-30a or PM-379, 20 nM) on the luciferase activities of the constructs containing the wild-type (wt-3′-UTR) or mutant-type 3′-UTR (mt-30a-3′-UTR or mt-379-3′-UTR) in OEC-M1 cells. The relative luciferase activity of each sample is measured at 48 h after transfection and normalized to Renilla luciferase activity. The data are represented as mean ± SD; ***p < 0.001 versus control mimics (PM-NC). **b** RT-PCR and Western blot analysis of DNMT3B level in SCC-15 and OEC-M1 cells after treatment with control mimics (NC), or miRNA mimics (PM-30a or PM-379, 20 nM) or miRNA inhibitor (AM-30a or AM-379, 20 nM) for 48 h. GAPDH and α-tubulin were used as internal control, respectively. **c** RT-PCR analysis of ADHFE1 and ALDH1A2 expression level after treatment with control mimics (NC, 20 nM), or miRNA mimics (PM-30a or PM-379, 20 nM) for 48 h in SCC-15 and OEC-M1 cells. GAPDH was used as internal control. **d** Relative miR-30a and miR-379 expression levels in 33 of adjacent normal tissues (N) compared with their own tumors (T). **e** Correlation analysis of DNMT3B and miR-30a or miR-379 in OSCC patients (*n* = 33) by qRT-PCR analysis. Pearson correlation coefficients and *p*-values were calculated as indicated. Red, tumor part; green, normal part

To test whether DNMT3B was targeted by miRNAs, the predicted miRNAs were retrieved from microRNA.org database combined with OSCC patients’ miRNA microarray data (GSE45238) [20]. MiR-30a and mi-379 were two of the potential candidates which with the high conservation of the putative binding sequences in the DNMT3B 3′-UTR (Additional file 2: Fig. S2). To test the hypothesis that miR-30a and miR-379 could direct target DNMT3B, we constructed dual-luciferase reporter plasmids containing either wilt-type or mutated 3′-UTR of DNMT3B for miR-30a or miR-379 (wt-3′-UTR, mt-30a-3′-UTR, mt-379-3′-UTR) (Additional file 2: Fig. S2). We observed a remarkable reduction of the wild-type 3′-UTR reporter activity in the presence of miR-30a or miR-379. In contrast, no obvious change in the mutant 3′-UTR reporter plasmid activity was observed (Fig. 3a). Transfecting OSCC cells with miR-30a and miR-379 mimics (PM) resulted in a significant repression of endogenous DNMT3B expression, conversely, depletion of miR-30a and miR-379 with miRNA inhibitor (AM) caused the upregulation of DNMT3B (at both mRNA and protein level)(Fig. 3b). Meanwhile, overexpression of miR-30a and miR-379 also increase ADHFE1 and ALDH1A2 mRNA expression in SCC-15 and OEC-M1 cells (Fig. 3c). We also analyzed the expression level of miR-30a and miR-379 in OSCC clinical samples. Results revealed that the levels of miR-30a and miR-379 were not only significantly reduced in tumors (Fig. 3d) but also with a strong negative correlation with the expression of DNMT3B (Fig. 3e). Taken together, our data suggested that miR-30a and miR-379 disturbed ADHFE1 and ALDH1A2 expression by direct targeting DNMT3B in OSCC.

### MiR-30a and miR-379 regulate OSCC cells proliferation through retinoid pathway

**Fig. 4:**
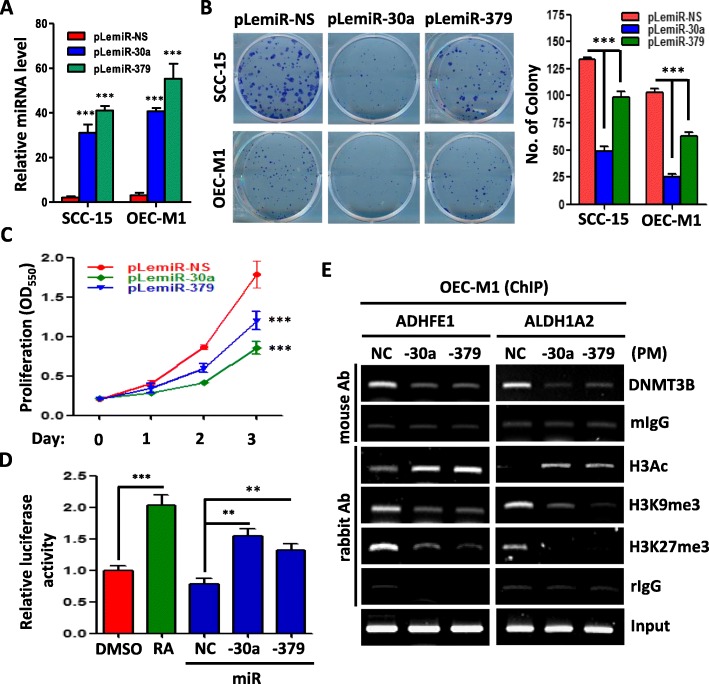
The effects of miR-30a and miR-379 overexpression on OSCC cells. **a** qRT–PCR analysis showing the expression level of miR-30a (pLemiR-30a) and miR-379 (pLemiR-379) compared with vector control (pLemiR-NS) in SCC-15 and OEC-M1 cells. **b** Colony formation assay after miR-30a and miR-379 transfection in SCC-15 and OEC-M1 cells for 7 days (left). The mean number of colonies for each well was determined from three independent assays (right). **c** Growth rates of OEC-M1 cells measured by MTT assay after vector control, miR-30a or miR-379 transfection. **d** OEC-M1 cells co-transfected with 1 μg of empty control vector and pRXR vector were incubated with the vehicle control (DMSO, 10 nM), 9-cis-RA (25 nM), control mimics (NC, 20 nM), miR-30a (20 nM) or miR-379 (20 nM). The relative luciferase activity of each sample is measured at 48 h after transfection and normalized to Renilla luciferase activity. **e** ChIP assay of ADHFE1 and ALDH1A2 promoter region was performed with OEC-M1 cells using anti-DNMT3B antibody, anti-acetyl-histone H3 (H3Ac) antibody, anti-histone H3 trimethylation of lysine 9 (H3K9me3) antibody, anti-histone H3 trimethylation of lysine 27 (H3K27me3) antibody, control mouse IgG (mIgG) antibody and control rabbit IgG (rIgG) antibody after treatment with control mimics (NC, 20 nM), or miRNA mimics (PM-30a or PM-379, 20 nM) for 48 h. All data are presented as mean ± SD; **p < 0.01; ***p < 0.001

To identify the tumorigenic roles of miR-30a and miR-379 in OSCC, we determined whether upregulation of these miRNAs could have effects on the viability of OSCC cells. Ectopic overexpression of miR-30a and miR-379 (Fig. 4a) decreased colony-forming ability in clonogenic proliferation assays (Fig. 4b) and reduced the growth rates of OSCC cells in MTT assays (Fig. 4c). Abundant evidence shows that retinoid inhibit cell-cycle progression and cell proliferation in a variety of human cancer cells through binding to RARs and RXRs heterodimers [26–28]. Moreover, miR-30a and miR-379 could increase ADHFE1 and ALDH1A2 expression and affect retinoid metabolic pathway (Figs. 2 and 3). To verify this hypothesis, RXR transcriptional activity was measured by the luciferase assay. We showed that overexpression of miR-30a and miR-379 significantly enhanced the RXR transcriptional activity relative to the control (NC) in OEC-M1 cells (Fig. 4d). Retinoic acid was used to stimulate RXR transcriptional activity and acts as a positive control (Fig. 4d). Furthermore, using chromatin immunoprecipitation (ChIP), we demonstrated that miR-30a and miR-379 treatment reduced the DNMT3B binding activity to ADHFE1 and ALDH1A2 promoter region in OEC-M1 cells. Simultaneously, we also found miR-30a and miR-379 treatment not only increased acetylation of Histone 3 (H3Ac), but also decreased trimethylation of Histone 3 lysine 9 (H3K9me3) and Histone 3 lysine 27 (H3K27me3) in ADHFE1 and ALDH1A2 promoter region (Fig. 4e). These results indicate that miR-30a and miR-379 inhibit OSCC cell growth by targeting DNMT3B to enhance ADHFE1 and ALDH1A2 activity.

### MiR-30a and miR-379 involved in arecoline and NNK induced epigenetic silencing

**Fig. 5:**
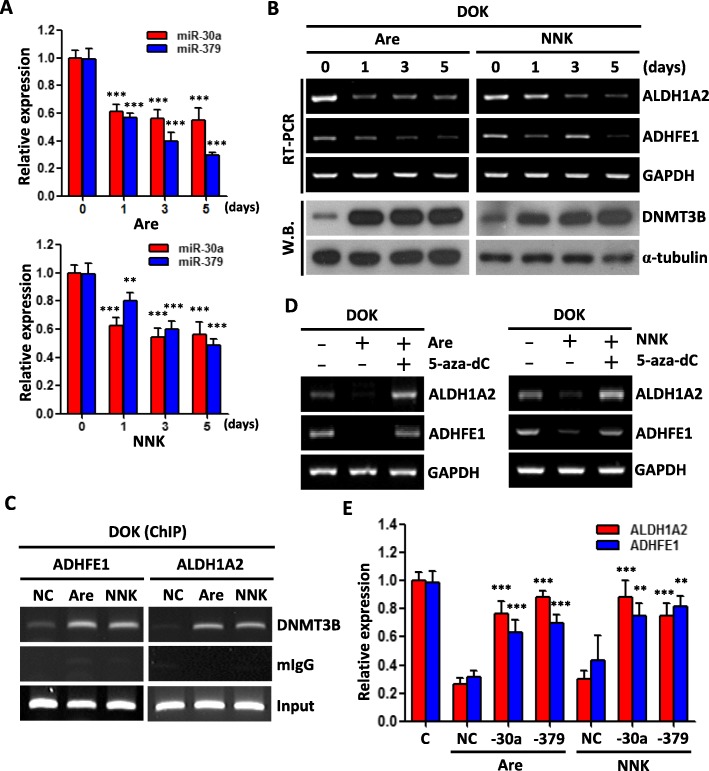
Arecoline and NNK induced DNMT3B activity and repressed ADHFE1, ALDH1A2 and miRNAs expression. **a** qRT–PCR analysis of miR-30a and miR-379 expression level after treatment with arecoline (50 μM) or NNK (10 μM) for indicated days. **b** RT-PCR analysis of ADHFE1, ALDH1A2 level and western blot analysis of DNMT3B level in DOK cells after treatment with arecoline (50 μM) or NNK (10 μM) for indicated times. GAPDH and α-tubulin were used as internal control. **c** ChIP assay of ADHFE1 and ALDH1A2 promoter region was performed with DOK cells using anti-DNMT3B antibody after treatment with vehicle control (DMSO, 10 nM), arecoline (50 μM) or NNK (10 μM) for 5 days. Mouse IgG (mIgG) antibody was used as negative control. **d** RT-PCR analysis of ADHFE1 and ALDH1A2 level in DOK cells after treatment with arecoline (50 μM) or NNK (10 μM) alone or combined with 5-aza-dC (5 μM) for 5 days. GAPDH was used as internal control. **e** qRT-PCR analysis of ADHFE1 and ALDH1A2 level in DOK cells after treatment with vehicle control (C), or arecoline (50 μM) plus control mimics (NC), miR-30a (20 nM), miR-379 (20 nM), or NNK (10 μM) plus control mimics (NC), miR-30a (20 nM), miR-379 (20 nM) for 5 days. GAPDH was used as an internal control. All data are presented as mean ± SD; **p < 0.01; ***p < 0.001

Smoking and betel quid chewing are two of the most important risk factors for oral cancer in Taiwan [19], and DNA hypermethylation has been reported to be related to smoking and betel quid chewing [18, 29]. Next, we attempted to determine the effect of arecoline, a major component of betel nut alkaloids, and 4-(Methylnitrosamino)-1-(3-pyridyl)-1-butanone (NNK), one of the major components of tobacco, on the expression of miR-30a, miR-379, DNMT3B, ADHFE1, and ALDH1A2. As shown in Fig. 5a, arecoline treatment significantly deceased the expression level of miR-30a and miR-379 in DOK cell. Notably, in these conditions, arecoline not only increased the expression level of DNMT3B, but also decreased the expression of ADHFE1 and ALDH1A2 (Fig. 5b). Using chromatin immunoprecipitation (ChIP), we demonstrated that arecoline treatment increased the DNMT3B binding activity to ADHFE1 and ALDH1A2 promoter (Fig. 5c). On the other hand, 5-aza-dC (DNMT inhibitor) treatment significantly rescued arecoline-repressed ADHFE1 and ALDH1A2 expression (Fig. 5d). In addition, miR-30a and miR-379 treatment significantly increased arecoline-repressed ADHFE1 and ALDH1A2 expression (Fig. 5e). Similar results were observed in the NNK-treated DOK cells (Fig. 5a-e). Taken together, these findings demonstrated that arecoline or NNK exposure could downregulate the miR-30a and miR-379 in oral cancer cells, consequently increase the DNMT3B protein level and recruit DNMT3B binding to ADHFE1 and ALDH1A2 promoter and caused DNA methylation.

### DNA methylation-silenced of ADHFE1 and ALDH1A2 is reversible

**Fig. 6:**
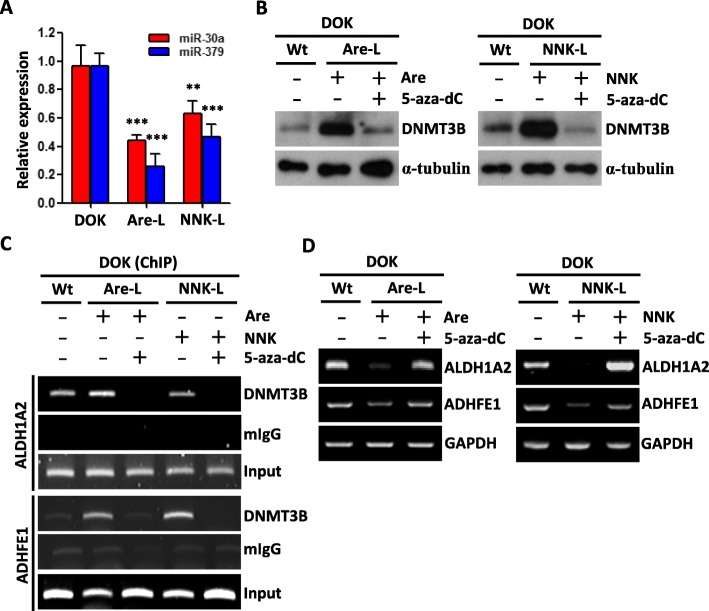
Effects of long-term treatment of arecoline and NNK. **a** Expression level of miRNAs in wildtype DOK cells and DOK cells long-term treatment with 50 μM of arecoline (Are-L) or 10 μM of NNK (NNK-L) for 12 months. The data are represented as mean ± SD; **p < 0.01; ***p < 0.001 versus wildtype DOK cells. **b** Western blot analysis of DNMT3B level in wildtype DOK cells (Wt), Are-L and NNK-L DOK cells after treatment with 5-aza-dC (5 μM) for 5 days. α-tubulin was used as internal control. **c** ChIP assay of ADHFE1 and ALDH1A2 promoter region was performed in Wt, Are-L and NNK-L DOK cells using anti-DNMT3B antibody after treatment with 5-aza-dC (5 μM) for 5 days. Mouse IgG (mIgG) antibody was used as negative control. **d** qRT-PCR analysis of ADHFE1 and ALDH1A2 level in Wt, Are-L and NNK-L DOK cells after treatment with 5-aza-dC (5 μM) for 5 days. GAPDH was used as internal control

Persistent smoking has lasting effects on DNA methylation, and methylation levels correlate with the cumulative dose of smoking [18, 30]. In order to assess the impact of long-term exposure of arecoline and NNK on DNA methylation and miRNA expression in this study, we generated the arecoline long-term treated DOK (Are-L) and NNK long-term treated DOK (NNK-L) for further assay. As shown, miR-30a and miR-379 exhibited lower expression in Are-L and NNK-L cells compared with wild-type DOK cells (Fig. 6a). Not only that, Are-L and NNK-L cells also have more DNMT3B protein amount and the stronger DNMT3B binding activity to ADHFE1 and ALDH1A2 promoter (Fug. 6b-c). As expected, the expression of ADHFE1 and ALDH1A2 were silenced in both Are-L and NNK-L cells (Fig. 6d). Conversely, 5-aza-dC-treated Are-L and NNK-L cells caused a significant DNMT3B repression and, consequently, caused a decrease in the DNMT3B binding activity to ADHFE1 and ALDH1A2 promoter and ADHFE1 and ALDH1A2 induction (Fig. 6b-d). These data suggest that persistent exposure of arecoline and NNK have a longer lasting effect on ADHFE1 and ALDH1A2 DNA methylation. Even though, DNA methylation can still reverse the expression of ADHFE1 and ALDH1A2 from NNK or arecoline long-term treated cells.

## Discussion

ADHFE1 and ALDH1A2 can participate in retinoid metabolism by oxidizing retinol to retinaldehyde to RA. RA binds a nuclear retinoic acid receptor and transcriptionally regulates genes involved in several biological processes involved in cell growth, differentiation, and carcinogenesis [8]. In the past, the promoter hypermethylation of ADHFE1 or ALDH1A2 was identified as a common issue in cancers and served as a risk factor with a poor prognosis [12, 13, 31, 32]. However, the epigenetic changes and the regulation mechanisms of ADHFE1 and ALDH1A2 genes in human OSCC remain unclear. Previously, we found a set of retinoid signaling related genes, including ADHFE1, ALDH1A2, CRBP1, PAX9, GDF10, TGFBR3, and PPARγ were frequently hypermethylated and downregulated in OSCC patient samples [7], suggesting the severe molecular defects in RA metabolism in oral cancer. RA signaling defects often cause resistance in solid tumors and result in RA treatment failure [15, 33]. Potential mechanisms of RA resistance in solid tumors have been proposed, such as the loss of RAR coactivators [34], impaired RAR signaling [15], increased RA metabolism [35] and decreased RA availability [36]. Besides that, RARβ is frequently lost early in carcinogenesis by epigenetic silenced, which is probably an important reason for the RA resistance in carcinoma [37]. However, the aberrant expression of RARβ is not observed in our cohort of OSCC patients (GSE37991). Instead of RARβ, our data demonstrate that epigenetic disruption of ADHFE1 and ALDH1A2 is a common event in human OSCC. ADHFE1 and ALDH1A2 catalyze irreversible steps in the synthesis of RA and thereby regulate distinct cellular functions [8]. It has been reported that cancer cells are unable to synthesize RA from retinol, due to loss of expression of ALDH6 [38, 39], and this conclusion is consistent with our findings. Our findings suggest the methylation-silenced expression of ADHFE1 and ALDH1A2 may be responsible for lower RA levels and, ultimately, to RA resistance in OSCC. In view of this, delivery of retinoids alone to patients treatment is challenging because of the rapid metabolism of retinoids in blood circulation as well as epigenetic changes can render cells RA resistant [40]. Therefore, the pharmacologic rationale is likely to require combination of retinoids and DNMT inhibitors for the treatment of OSCC patients which lack of ADHFE1 and ALDH1A2 expression.

Interestingly, aberrant DNA methylation has been reported to be involved in oral cancer associated with tobacco smoking and betel quid chewing [41, 42]. Tobacco smoking and betel quid chewing are the most common environmental risk factors for the development of oral cancer in Taiwan [43]. In this study, we found that NNK and arecoline treatment could recruit DNMT3B to ADHFE1 and ALDH1A2 promoter region, subsequently repressed the expression of ADHFE1 and ALDH1A2 in oral cancer. Epidemiological studies reveal that epigenetic modifications, such as DNA methylation, may present a biological biomarker of lifetime accumulation of environmental exposures related to aging [44], alcohol [45], smoking [18, 46], and perhaps many others. Here, our experiments demonstrated that long-term exposure of NNK and arecoline has lasting effects on DNA methylation, especially on promoter region of ADHFE1 and ALDH1A2. Notably, DNMT inhibitor treated OSCC cells, which were long-term exposure of NNK and arecoline, caused a significant repression of DNMT3B and dramatic re-expression of ADHFE1 and ALDH1A2, suggesting that lasting effects on DNA methylation by NNK and arecoline are reversible. These results highlight the lack of ADHFE1 and ALDH1A2 expression could serve as an attractive biomarker to stratify OSCC patients and may be better to use a combination of retinoids and demethylating agents for therapeutic/preventive strategies in patients with oral cancer.

In addition, we also found that the reduced miR-30a and miR-379 can lose an inhibitory effect on DNMT3B, which contributes to hypermethylation of the ADHFE1 and ALDH1A2 genes. It has been reported that miR-30a and miR-379 are downregulated in many cancers and act as a tumor suppressor to regulate various biological processes, including proliferation, cell cycle, apoptosis, and metastasis [47, 48]. The present study showed that miR-30a and miR-379 can downregulate DNMT3B which in turn inhibit methylation in the promoter of the ADHFE1 and ALDH1A2 genes leading to higher ADHFE1 and ALDH1A2 expression and growth inhibition. Therefore, we identified that miR-30a and miR-379 have anti-proliferation ability through their effect on the increase of ADHFE1 and ALDH1A2 expression. Thus, it is advised that miR-30a and miR-379 could be potentially regarded as promising biomarkers in oral cancer development and progression. Although the detail mechanisms of downregulation of miR-30a and miR-379 in oral cancer remain unclear, tobacco smoking and betel quid chewing seem to play a role in regulating these miRNAs expression.

## Conclusion

In summary, the results of the current study demonstrate that tobacco smoking and betel quid chewing could repress miR-30a and miR-379, which upregulate the DNMT3B expression, in turn, lead to the hypermethylation of ADHFE1 and ALDH1A2 genes, consequently, promote the oncogenic activity. Our findings implicate ADHFE1 and ALDH1A2 as tumor suppressor genes in oral cancer and provide rationale for further investigation of retinoids in combination with epigenetic modifiers for the prevention or treatment of oral cancer.

## Supplementary information

**Additional file 1: Table S1.** List of primer sequences.

**Additional file 2: Figure S1.** Expression level of DNMTs in OSCC tissues. **a** Microarray analysis of DNMT1, DNMT3A and DNMT3B expression levels in OSCC tumors (*n* = 40) compared with their own adjacent normal tissues or compared with patients’ stage. Expression levels are expressed as the log2 ratios. **b** Correlation analysis of DNMT1, DNMT3A and DNMT3B with ADHFE1 or ALDH1A2 in human OSCC patients (*n* = 40). Each spot indicates the value of Tumor/Normal ratio. **Figure S2.** Schematic representation of the putative miR-30a (*a*) and miR-379 (**b**) binding sequence in the 3′-UTR of DNMT3B with wild-type form (wt-3′-UTR) and mutant form (mt-30a-3′-UTR or mt-379-3′-UTR). The mutated nucleotides are labeled black color with underline.

## Data Availability

The dataset supporting the conclusions of this article is included within the article.

## Abbreviations

3′-UTR: 3′-Untranslated region
5-aza-Dc: 5-aza-2-deoxycytidine
ADHFE1: Alcohol dehydrogenase, iron containing 1
ALDH1A2: Aldehyde dehydrogenase 1 family, member A2
ChIP: Chromatin immunoprecipitation
DNMT3B: DNA methyltransferase 3B
GAPDH: Glyceraldehyde 3-phosphate dehydrogenase
IHC: Immunohistochemistry
miRNAs: microRNAs
MTT: 3-[4,5-dimethylthiazol-2-yl]-2,5-diphenyl tetrazolium bromide
NNK: 4-(Methylnitrosamino)-1-(3-pyridyl)-1-butanone
OSCC: Oral squamous cell carcinoma
RA: Retinoic acid
qRT-PCR: Quantitative real-time PCR
RT-PCR: Reverse transcription PCR
