# Supplementary material for: MiR-30a and miR-379 modulate retinoic acid pathway by targeting DNA methyltransferase 3B in oral cancer
Source: J Biomed Sci. 2020 Apr 2;27:46. doi: 10.1186/s12929-020-00644-z (PMC7114797; doi:10.1186/s12929-020-00644-z)
Supplement: Supplementary file 1 — Additional file 1: Table S1. List of primer sequences. [file 12929_2020_644_MOESM1_ESM.pdf]

Table-S1. List of primer sequences.

Primer Name	Sequence
Gene Expression Primers	
GAPDH-F	GAAGGTGAAGGTCGGAGT
GAPDH-R	GAAGATGGTGATGGGATTTC
DNMT1-F	CCCCTGAGCCCTTACCGAAT
DNMT1-R	CTCGCTGGAGTGGACTTGTG
DNMT3A-F	GACAGAGGCACCGTTCAC
DNMT3A-R	AGGGCATGGCGGGCATCT
DNMT3B-F	CCCGACTCTTACCTTACCATCG
DNMT3B-R	GGTCCCCTATTCCAAACTCCT
ADHFE1-F	TGCTGGTGTTCATCTGTGCCAT
ADHFE1-R	TTCCGGAGCGTGTCTGCCAAC
ALDH1A2-F	GGATGACCATTCCTGTAGATGGAGA
ALDH1A2-R	GCTATTGCTGCCCCAGCCGTTG
miRNA Expression Primers	
Universal Reverse	GTGGAGGGTCCGAGGT
RNU-44-RT	GTTGGCTCTGGTGCAGGGTCCGAGGTATTCGCACCAGAGCCAAC AGTCAG
RNU-44-F	GCCCTGGATGATGATAGCAA
miR-30a-RT	GTTGGCTCTGGTGCAGGGTCCGAGGTATTCGCACCAGAGCCAAC CTTCCA
miR-30a-F	CCGCTTGTAAACATCCTCGAC
miR-379-5p-RT	GTTGGCTCTGGTGCAGGGTCCGAGGTATTCGCACCAGAGCCAAC CCTACG
miR-379-5p-F	GGGTGGTAGACTATGGAA
Cloning Primers	
DNMT1-3’UTR-F	CCGCTCGAGGGAAGCTGCTAAGGACTAGTT
DNMT1-3’UTR-R	GCTCTAGAGGTTTATAGGAGAGATTTATTTGAAG
DNMT3B-3’UTR-F	CCGCTCGAGGACTACTTTGCATGTGAATAG
DNMT3B-3’UTR-R	GCTCTAGAAGACAAATACTGATTTTAATTAAAC
DNMT3B-3’UTR-Mt-miR379-F	CCCTTGATGGGCGTACCACTCAGAGAAACAATGGCTAAGATACC A
DNMT3B-3’UTR-Mt-miR379-R	GTGGTACGCCCATCAAGGGAAAAAAAAAAAGCCACGTTGCTAC CTAC
DNMT3B-3’UTR-Mt-miR30a-1F	TACTGCTCTGCCTCTACAGACGTGTGCAGTTGTAGGCATGTAGC
DNMT3B-3’UTR-Mt-miR30a-1R	CTACAACTGCACACGTCTGTAGAGGCAGAGCAGTAGCCAGTTTT C
DNMT3B-3’UTR-Mt-miR30a-2F	CTATTTTTAGCTCTAACGTTTTCATTAAAATTTTTTTTGTAACTGG
DNMT3B-3’UTR-Mt-miR30a-2R	GAAAACGTTAGAGTTAAAAATAGAAGTTTGAGATTTTAAAAAGT G
DNMT3B-3’UTR-Mt-miR30a-3+4F	TAGAGCTCACCTTATGCTCAATTAAAATCAGTATTTGTCTTCTAGA GTC
DNMT3B-3’UTR-Mt-miR30a-3+4R	TAATTGAGCATAAGGTGAGCTCTAGGCATCCGTCATCTTTCAG
