# Supplementary figures and images for: MiR-30a and miR-379 modulate retinoic acid pathway by targeting DNA methyltransferase 3B in oral cancer
Source: J Biomed Sci. 2020 Apr 2;27:46. doi: 10.1186/s12929-020-00644-z (PMC7114797; doi:10.1186/s12929-020-00644-z)

Figure-S1

A

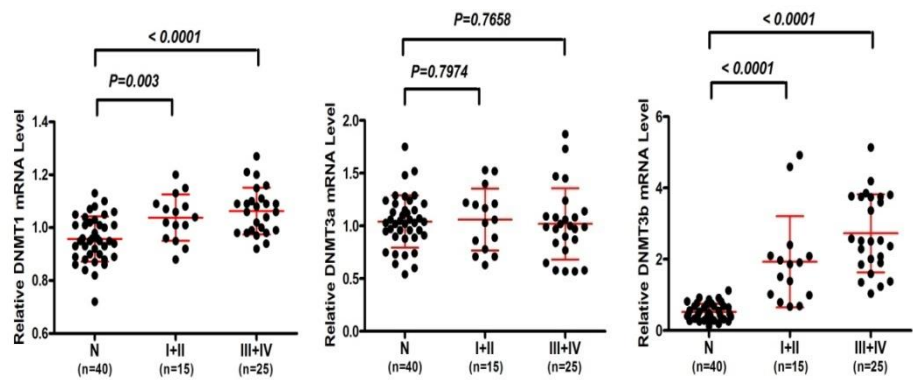

B

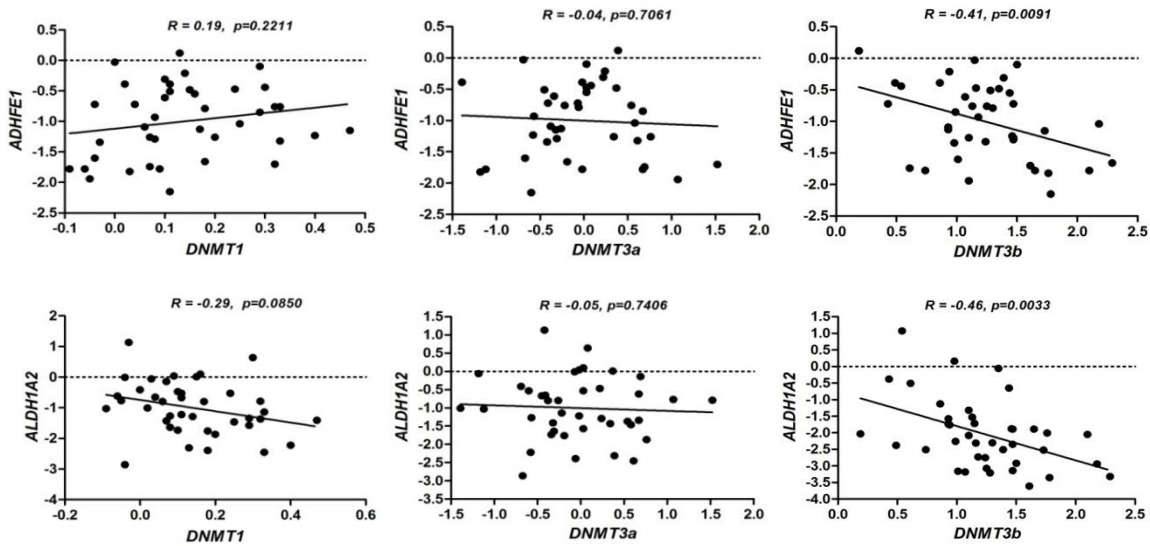

Figure-S2

A

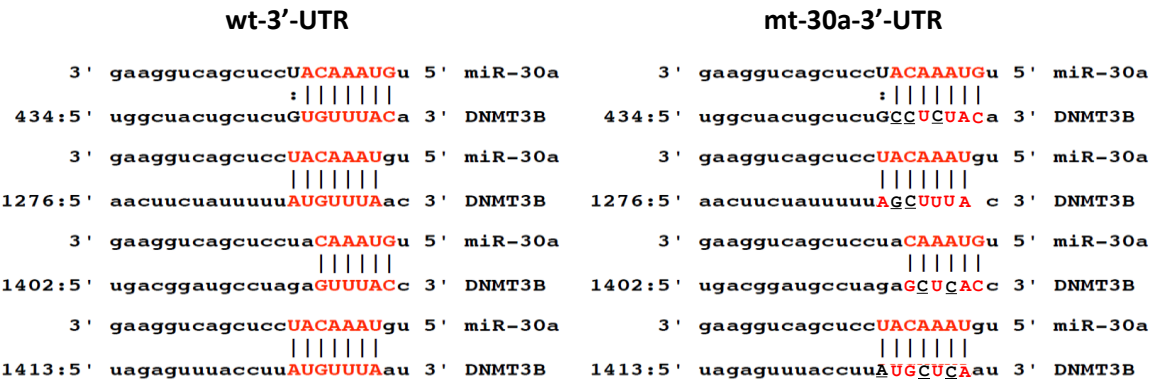

B

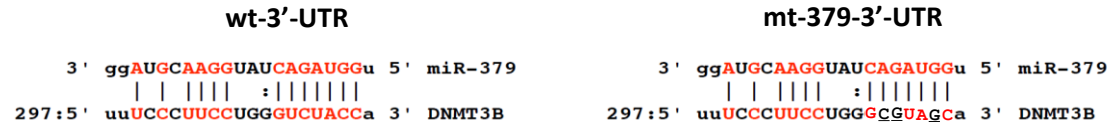

Supplement: Supplementary file 2 — Additional file 2: Figure S1. Expression level of DNMTs in OSCC tissues. a Microarray analysis of DNMT1, DNMT3A and DNMT3B expression levels in OSCC tumors (n = 40) compared with their own adjacent normal tissues or compared with patients’ stage. Expression levels are expressed as the log2 ratios. b Correlation analysis of DNMT1, DNMT3A and DNMT3B with ADHFE1 or ALDH1A2 in human OSCC patients (n = 40). Each spot indicates the value of Tumor/Normal ratio. Figure S2. Schematic representation of the putative miR-30a (a) and miR-379 (b) binding sequence in the 3′-UTR of DNMT3B with wild-type form (wt-3′-UTR) and mutant form (mt-30a-3′-UTR or mt-379-3′-UTR). The mutated nucleotides are labeled black color with underline. [file 12929_2020_644_MOESM2_ESM.pdf]
